# Analysis of the Influence of Sports on College Students' Music Vocalization Training Based on Monte Carlo and Dynamic Adjustment Factor Algorithm

**DOI:** 10.1155/2022/3443404

**Published:** 2022-07-16

**Authors:** Li Hua, Lin Yu

**Affiliations:** ^1^Jiangxi University of Technology, Nanchang 330098, China; ^2^Adamson University, Manila 0900, Philippines; ^3^SEGi University, Kuala Lumpur 47810, Malaysia

## Abstract

With the continuous improvement of people's living standards, the requirements of music majors for their training standards are also increasing, which leads to the development of music training in the direction of intelligence. This paper discusses the problems of breathing, coordination, and muscle control ability in vocal training and puts forward a vocal training method based on dynamic adjustment factor and Monte Carlo algorithm to solve the difficult problem of vocal training for college students and understand the relationship between vocal training and exercise. Firstly, the sports training set and vocal pronunciation training set are constructed in the form of clustering, and the samples in the set are analyzed discretely to ensure that the samples conform to normal distribution; then, using the Monte Carlo algorithm analyzes the two sample sets and finds out the relationship between exercise and vocal training. Finally, according to breathing, coordination, muscle control ability, and other indicators, calculate the impact of exercise on vocal sound. MATLAB simulation shows that the method proposed in this paper can analyze the influence of exercise on vocal vocalization from the perspective of breathing, coordination, muscle control ability, and other indicators. The accuracy of judgment results is more than 95%, and the time is less than 1 min. All indicators are better than traditional vocal training methods (90%, 2 min), which shows the effectiveness of the method proposed in this paper.

## 1. Introduction

With the development of kinematics theory and biological theory, vocal training of vocal music majors is more reasonable, scientific, and intelligent. However, under the condition of lack of vocal training experience, college students will be unable to master vocal skills. The key to vocal training is the control of breath and pharyngeal muscles, that is, breathing, coordination, and muscle control ability [1]. Breathing, coordination, and muscle control can be improved by mastering skills and exercising. How to make a reasonable way of exercise and find out the relationship between exercise and vocal vocalization is a problem that college students need to solve in vocal music majors at present. Some scholars believe that the classification of exercise and vocal training makes the research results more accurate. However, the complexity of exercise indicators and students' lack of mastery of vocal skills make the training of vocal skills more complicated. Some scholars believe that the premise of vocal training is the speaker's physical condition, so we should improve our vocal skills by mastering breath, muscles, and time. Therefore, some scholars put forward a variety of exercise indicators [2], such as breath, laryngeal position, abdominal strength, and the synergy of related muscles. The specific results are shown in Table 1.

Some scholars have a theoretical analysis of vocal training methods and think that the premise of vocal training is of great significance to improve the training level [3]. However, there is a lack of quantitative analysis on the influence degree of respiration, coordination, and muscle control ability. Based on this, this paper puts forward a vocal training model based on the Monte Carlo algorithm, which can improve the vocal theory and improve the professional quality of college students. From the above analysis, we can see that there are many types of research on the Monte Carlo algorithm at home and abroad, but there are few types of research on its application in sports exercise and music training. At present, music training focuses more on comprehensive analysis methods, looking for an intelligent judgment and analysis method to guide students in music training. Monte Carlo algorithm can be used to analyze the indexes in music training and realize the deepening and dynamic research of the indexes, which meets the current requirements. Due to the increasing requirements of music training, the research and analysis methods of training are developing in the direction of diversification, pluralism, and intelligence. Compared with a genetic algorithm, the Monte Carlo algorithm has the characteristics of comprehensiveness, intelligence, and diversification, which is more suitable for continuous dynamic analysis and can meet the requirements of diversified vocal training. At present, the Monte Carlo algorithm has a wide range of applications, gradually penetrated into the field of music, and merged with other algorithms, effectively promoting the development of vocal training. Monte Carlo algorithm has strong advantages, which can analyze the data continuously and find the potential problems and relationships in the data. At the same time, the Monte Carlo algorithm also has some shortcomings, which cannot analyze massive data, and needs to be combined with other methods. Therefore, the Monte Carlo algorithm has a wide range of applications, which can better carry out data analysis and research. Therefore, the Monte Carlo algorithm meets the current research needs and has research feasibility.

## 2. Related Concepts

### 2.1. Vocal Theory

Vocal vocalization theory is to use a variety of vocalization skills to adjust the vocalization position, breath time, and muscle strength of college students. Vocalization theory requires college students to use a variety of skills to improve their vocalization level [4]. In the mid-20th century, abdominal vocalization, bone vocalization, and other skills, as well as vocalization posture, were widely used in vocalization training. At present, vocalization theory holds that vocalists should use comprehensive vocalization skills, including sports, to improve college students' vocalization levels. The Monte Carlo algorithm can comprehensively analyze the data of exercise, mainly to analyze the relationship between the data and the internal attributes of the data and reveal the internal relationship between the data more deeply. Although the Monte Carlo algorithm can be used for deep-level data mining, it cannot be used for continuous data analysis and cannot show the internal relations between data. A dynamic factor algorithm can quantify physical exercise and music training, establish the dynamic relationship between them, and analyze the data continuously. Therefore, the combination of the Monte Carlo algorithm and dynamic factors can realize the interaction between different algorithms, dig into the connection between sports exercise and music training more deeply, and guide music training to be carried out orderly. To further study the theory of vocalization by Fujisawa et al. [5], this paper puts forward a quantitative analysis method, which integrates the technical indicators of vocalization into training and forms a comprehensive combination of vocalization skills to improve the overall level of vocalization.

**Theorem 1 .**

Suppose that the research set *T*=(*d*_*i*_∩*b*_*i*_*|i*=nature), any exercise content *d*_*i*_ ∈ *n*, is the result of vocal training *b*_*i*_ ∈ *n*. The effect result of exercise on vocal training is L (d_i_, b_i_, n) and the process sub-function is K (d_i_, b_i_, n). If the combination of exercise is *D*_*i*_, the relationship function between exercise and vocal training can be obtained, as follows:

(1) $$\begin{array}{c} D_{i}=K{\left(d_{i},b_{i},n\right)}_{T}\cdot L\left(d_{i},b_{i},n\right), \end{array}$$

where *n* is a natural number. If *w* is the standard of vocal training and *λ* is the score of vocal evaluation, when the vocal training is optimal, the sports training content is the most.

**Theorem 2 .**

If the deviation coefficient *ξ* of the analysis result is larger [6], the normal analysis should be carried out on the initial data set, and the specific analysis process is

(2) $$\begin{array}{c} T=\left(d_{i}\cap b_{i}|\right)⟶N\left(n,\sigma^{2}\right). \end{array}$$

Among them, *σ*^2^ is the variance of deviation coefficient, which mainly reflects the deviation degree of analysis results.

**Theorem 3 .**

If exercise affects the result of vocal training *K*(*d*_*i*_, *b*_*i*_)=*φ*(*d*_*i*_)′*φ*(*b*_*i*_)+*φ*(*d*_*i*_)*φ*(*b*_*i*_)′, then the process component function *L*(*d*_*i*_, *b*_*i*_, *n*) can be expressed as

(3) $$\begin{array}{c} L\left(d_{i},b_{i},n\right)=\ln\left[K\left(d_{i},b_{i}\right)\right]. \end{array}$$

Among them, ln[·] belongs the extended function, which ensures the stability of calculation results.

From formulas (1)∼(3), we can see that the process analysis function *L*(*d*_*i*_, *b*_*i*_, *n*) in the vocal theory and the verification of the initial data set are the key points of this paper.

### 2.2. Improved Vocal Training Methods

#### 2.2.1. Traditional Vocal Training Methods

Vocal training methods are mainly based on college students' age, gender, BMI, grade, vocal types, and comprehensive analysis of college students' vocal training [7]. During initialization *T*=(*d*_*i*_∩*b*_*i*_*|i*=nature), the exercise training content is the same, and the breathing, coordination, and muscle control ability are comprehensively analyzed [8]. Firstly, the sports content is randomly selecte and optimized in the sports exercise content with better evaluation results, and the optimal combination of sports schemes is obtained by comparison, which is determined by the least square method. Then, using the strategy of roulette to randomly compare the contents of sports and exercise and carry out corresponding standard evaluation, the sports contents are arranged according to the scoring results, and the final results are obtained, as shown in Table 2.

Assuming that the relationship between exercise content and vocal training results is 1 : n, and the selection of exercise content is random [9], and *R*=(*d*_*i*_, *b*_*i*_) expression is used as the process function between performance, exercise content, and results can be expressed as

(4) $$\begin{array}{c} L_{i}\left(d_{i},b_{i}\right)=R\left(d_{i},b_{i}\right)\cdot K\left(d_{i},q_{i},b_{i}\right)+\xi . \end{array}$$

Among them, *d*_*i*_ and *b*_*i*_ are a random combination, in which *ξ* is the adjustment coefficient of the combination, and *R*(*d*_*i*_, *b*_*i*_) is a relational function. If the coefficient is close to 1, it shows that the result is more accurate. Iterative technology of sports exercise content and cross combination of different contents [10] realize the update of sports exercise content. Under the constraint condition, the combined form of sports content is analyzed by ranking combination form, and the specific results are

(5) $$\begin{array}{c} d_{i}=\frac{w\cdot b_{i}!}{\lambda \cdot \left(n-d_{i}\right)!}. \end{array}$$

Among them, *b*_*i*_ ∈ [0, *n*/2].

The auxiliary scheme *d*_*j*_ is to combine the sports exercise contents with probability *p*_*i*_ to obtain the optimal sports exercise contents. The combination process of sports exercise contents is

(6) $$\begin{array}{c} d_{j}=\frac{p_{i}}{C_{n}^{n-p}}. \end{array}$$

*p* is the probability sum of the combined contents. If the exercise content has not got the optimal solution after infinite iterations, the optimization of the exercise content will be abandoned and the combination will be combined with the auxiliary scheme, that is, *d*_*i*_ · *d*_*j*_.

#### 2.2.2. Dynamic Adjustment of Monte Carlo Algorithm

In the analysis of sports training and vocal training, the exercise program cannot guarantee the minimum adjustment coefficient [11], and there will be more subjective judgments, which will reduce the accuracy of vocal training results. Therefore, in the process of choosing sports training content, we should try our best to expand the number of sports training content and constantly adjust the sports training content. When judging the content of a single exercise, linear parameters *ρ* can be set to make it present normal distribution and reduce the randomness of exercise content. To avoid excessive subjectivity, this paper introduces dynamic factors *ν*, and its dynamic adjustment strategy is

(7) $$\begin{array}{c} \rho =\min i\nu_{i}^{\prime }. \end{array}$$

Among them, *ν*_*i*_ is I adjustment. Exercise content and vocal training affect the update of results, which can be expressed as follows:

(8) $$\begin{array}{c} \Delta L\left(d_{i},b_{i}\right)=w\cdot K\left(d_{i},b_{i}\right)+\nu_{i}T\left(d_{i},b_{i}\right). \end{array}$$

**Figure 1:**
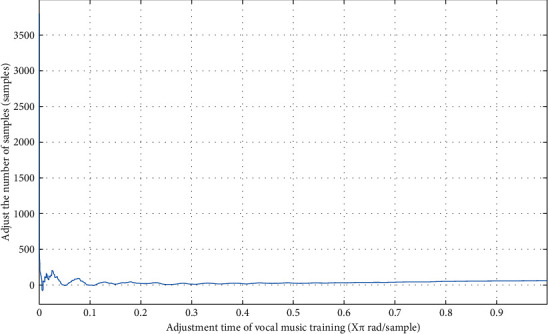
Dynamic adjustment of exercise content to vocal training.

From formula (8), it can be seen that when the initial adjustment is made, *ν* and*w* are relatively large [12], its purpose is to expand the scope of exercise content and keep the diversity of exercise content as much as possible. In the later adjustment stage, *ν* and*w* are relatively small, and the judgment range is continuously reduced to improve the accuracy of the calculation results. The results are shown in Figure 1.

As can be seen from Figure 1, the analysis process of the influence of exercise on vocal training belongs to the process of expanding the scope first, then shortening the scope, and constantly adjusting the sports content. This shows that the dynamic adjustment of exercise can make the exercise content meet the requirements and improve the accuracy of vocal training results [13].

#### 2.2.3. Introduction of Comprehensive Factors

When certain exercise content is collected many times and reaches the mining limit, the leading scheme will be changed into an adjustment scheme, looking for new exercise content and combining new solutions of strategies. Since the vocal music training method is related to personal physique and learning awareness, a “subjective training program” will be adopted in the training process. The probability of a “subjective scheme” in exercise content is high, and it is positively correlated with the complexity of exercise content. To make up for the above shortcomings, this paper introduces comprehensive factors *f*(*x*_*i*_) to reduce the complexity of exercise content by the probability density method and help avoid a “subjective scheme.” The introduction formula of comprehensive factors is

(9) $$\begin{array}{c} f\left(x_{i}\right)=\frac{F\left(b_{i},d_{i}\right)}{\theta {\sum_{i=1}^{n}F\left(\Delta d_{i},\Delta b_{i}\right)}^{2}}. \end{array}$$

When *F*(*b*_*i*_, *d*_*i*_)=1, the content of *F*(*b*_*i*_, *d*_*i*_) middle exercise obeys the normal distribution Cauchy (0, 1). Moreover, at that time,*θ*=2*π*, and the complexity of representing exercise content was the highest; otherwise, the complexity of *θ*=0 was the lowest. In addition, the motion content of Cauchy (0, 1) is smaller than that of Gauss (0, 1), which can reduce the influence of subjective factors in the function.

### 2.3. Analysis of the Influence of Vocal Training

#### 2.3.1. Vocal Training Model Based on Monte Carlo Algorithm

A reasonable analysis of college students' vocal training is the main purpose of measuring this training method [14]. The relationship between sports training content vocal training standard sports training content and vocal training standard and comprehensive adjustment factors is dynamic and should be adjusted constantly to improve the accuracy of the analysis. From formula (8), it can be seen that, in the initial judgment of exercise content, the scope of exercise content should be continuously expanded, and in the later judgment, attention should be paid to the judgment of breathing, coordination, muscle control ability, and other indicators, so different exercise contents should be based on corresponding evaluation standards. The application scope of the Monte Carlo algorithm is different, and its strategies are also different. To better analyze the Monte Carlo algorithm, find out the influencing factors of occurrence training, better conduct occurrence training, and guide music majors to exercise, the Monte Carlo algorithm should be constrained. The Monte Carlo algorithm should choose the corresponding strategy according to the deepest situation. Therefore, according to different indicators, we should formulate corresponding vocal training strategies.

(1) The vocal training strategy for breathing is
  (10) $$\begin{array}{c} F\left(x_{i}\right)=\alpha \cdot F\left(d_{i},b_{i}\right)+\beta \cdot F\left(d_{i},b_{i}\right), \end{array}$$
*α* is the breath time coefficient, and *β* is the breath quantity coefficient.
(2) The vocal training strategy in coordination is
  (11) $$\begin{array}{c} F\left(x_{i}\right)=a\cdot F\left(d_{i},b_{i}\right)+b\cdot F\left(d_{i},b_{i}\right), \end{array}$$
*a* is the coordination coefficient of breathing, and *b* is the muscle coordination coefficient.
(3) Vocal training strategies for muscle control is
  (12) $$\begin{array}{c} F\left(x_{i}\right)=A\cdot F\left(d_{i},b_{i}\right)+B\cdot F\left(d_{i},b_{i}\right). \end{array}$$
*A* is the time coefficient of exertion, and *B* is the coefficient of exertion. Given the above three aspects, adjust the sports content. In this paper, the improvement of vocal training methods: on the one hand, adjust the sports content, set the vocal training standards*w*, the corresponding scores*λ*, and according to the above four strategies for random selection, to achieve multiple analyses of sports training content. In the later period of exercise content adjustment, gradually strengthen the depth of research, realize the diversity of vocal training, and improve the comprehensiveness of analysis results [15]. On the other hand, balance the global judgment and local judgment ability of vocal training and integrate inertia factor Δ*ν*_*i*_ and moderate function *Z*(*d*_*i*_, *b*_*i*_) to realize the best fusion of exercise content and training effect.

#### 2.3.2. Monte Carlo Algorithm Is Used to Judge the Data Set Dynamically

The data set is the main object of dynamic optimization. The vocal training method in this paper is based on clustering dynamic optimization and constructs dynamic judgment of dynamic data. Different sports content uses different dynamic adjustment factors, corresponding parameters, and data elimination. The sports content and vocal training set are randomly divided into different subsets, and each subset chooses different strategies. In each calculation process, the subset will be evaluated by different standards. After each exercise content is collected, the corresponding complexity and fitness will be calculated, and the best vocal training scheme will be recorded. The optimal aggregation of other subsets can improve the effect of vocal training.

### 2.4. Calculation of the Final Vocal Training Results

**Figure 2:**
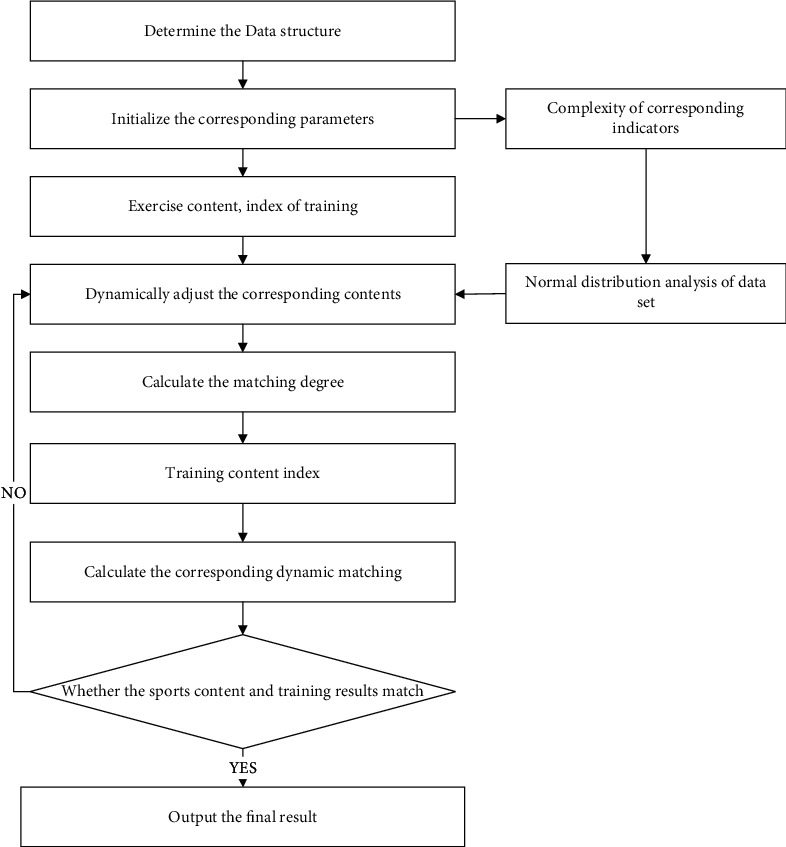
Steps for improving the vocal training method.

The basic idea of the dynamic vocal training method is to use a variety of strategies to analyze the complex exercise content and combine the results of breathing [16], coordination, and muscle control ability to obtain the global optimal training scheme and adjust the corresponding complexity. The implementation steps of the vocal training method in this paper are shown in Figure 2.

**Step 1 .**

Determine the structure and complexity of data and determine the structure distribution and complexity of exercise content.

**Step 2 .**

Initialize the exercise content, training index, and calculated iteration times *n* = 100, Δ*ν*_*i*_ ∈ [0,0.9].

**Step 3 .**

Build the fitness function. Randomly generate exercise sets, select corresponding training strategies and training standards *w*, and evaluate scores *λ*. According to the complexity *w*=0.22 and *λ*=0.51. Judge the normality of the data set, and choose the best scheme Mobley et al. [17].

**Step 4 .**

Combine global and local optimal matching of strategies. The initial set is divided into five subsets, and the fitness and fitting value are calculated to get the optimal global training result and the training set of each subset.

**Step 5 .**

Iteration of exercise content and training effect. According to the change of exercise content, 5 subsets dynamically adjust factors, incorporate adjustment factor *C* and inertia standard Δ*ν*_*i*_.

**Step 6 .**

Dynamic optimization of each subset. After adjusting the exercise content, the global optimal training scheme is calculated, and the optimal analysis is carried out with relevant standards.

**Step 7 .**

Judge whether the exercise content reaches the best match and whether the iteration times reach the maximum. If steps 1∼5 are repeated, the analysis will be stopped.

## 3. Empirical Analysis

### 3.1. Performance Analysis of Monte Carlo Algorithm

To further verify the performance of dynamic vocal training methods, the training evaluation indexes are analyzed, which are breath, coordination, strength, and final results. The performance analysis process is as follows:

(1) Breath judgment function: the result is
  (13) $$\begin{array}{c} f_{1}\left(x\right)=\sum_{i=1}\frac{b_{i}^{2}}{10}\cdot \;\cos\left(2\pi d_{i}\right)+\xi . \end{array}$$
(2) Coordination judgment function: the result is
  (14) $$\begin{array}{c} f_{2}\left(x\right)=\sum_{i=1}d_{i}^{2}+\xi . \end{array}$$
(3) The strength judgment function: the result is
  (15) $$\begin{array}{c} f_{3}\left(x\right)=\frac{d_{i}}{50}+\underset{x⟶0}{\lim}b_{\partial i}^{\sqrt{1/n}}. \end{array}$$
(4) The final result judgment function: the result is
  (16) $$\begin{array}{c} f\left(x\right)=1+\sum_{i=1}\frac{f_{i}^{2}}{100}, \end{array}$$
where *x* ∈ [0,100], *i* is 4. In this paper, through the relevant parameters set, the total number of exercise content is 20 items, the number of iterations is 50, the maximum inertia scheme Δ*ν*_max_=2.5, and the optimal inertia scheme Δ*ν*_min_=0.3. The results of the four test functions are shown in Table 3.

**Figure 3:**
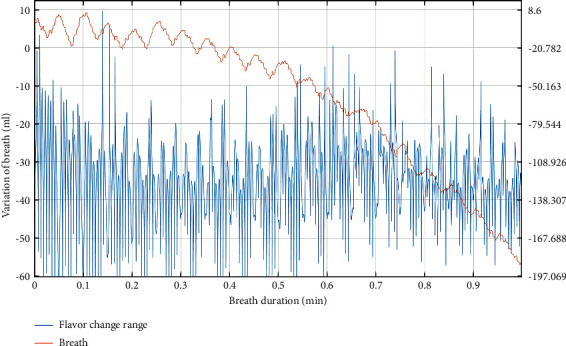
Vocal training effect of the breath test.

**Figure 4:**
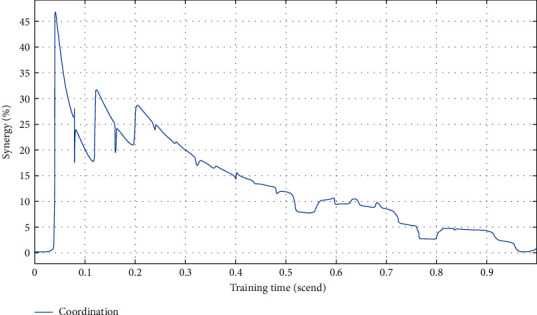
Vocal training effect of the coordination test.

**Figure 5:**
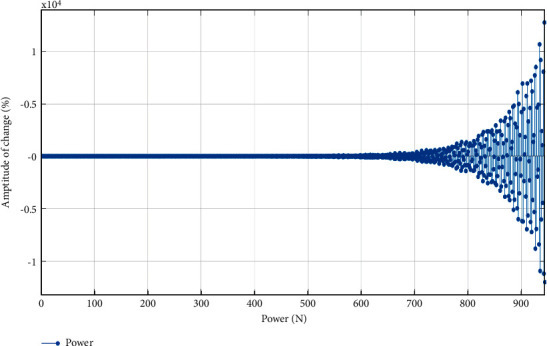
Coordination test effect of the strength test.

**Figure 6:**
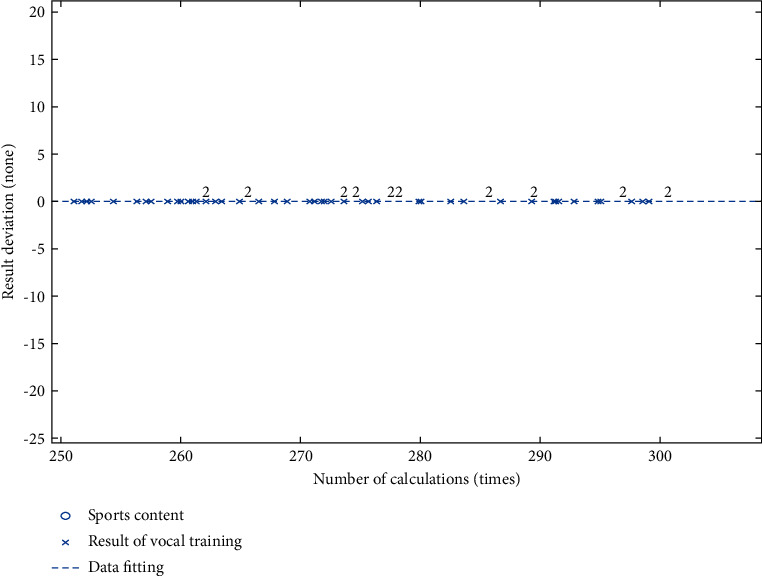
Overall training effect of vocal music training.

From Table 3, we can see that the improvement of vocal training methods is better in terms of breath, coordination, and strength, and the final result is also better. Moreover, the overall optimization degree and the degree of meeting the standard of the improved vocal training method are more than 90%. To analyze more concretely, different sports contents are simulated to verify the effect of vocal training, as shown in Figures 3–6.

Through the analysis in Figure 3, it can be seen that sports training can effectively improve the rhythm and frequency of breathing and improve the overall effect. In addition, exercise training can increase the amount of breathing, so that the sound can last longer.

As can be seen from Figure 4, sports training can increase the coordination of occurrence. In the early stage, coordination and fluctuation changes occurred, while in the later stage, the changes were relatively stable.

The overall strength effect is better, and the strength increases continuously in the later period, which shows that sports training can improve vocal strength.

It can be seen from Figures 3∼6 that the selection of the best scheme of dynamic vocal training method is faster and more stable, which is better than the vocal training method. Therefore, the dynamic vocal training method in the combination of strategy speed, combination of strategy accuracy performance is better, and the combination of the strategy process is more stable [18].

### 3.2. Overall Effect of Vocal Training

Based on skipping rope, sprinting, push-ups, and other sports, this paper carries out vocal training in folk music, bel canto, popular music, and rock music and judges the overall effect of vocal training on college students. Because the observation time is 1 month, some observation data are missing, so the previous observation data are supplemented to ensure the integrity of the data. The specific contents are shown in Table 4.

From Table 4, we can see that the training scores and training results of the observation group are better than those of the control group, and the training results are one grade higher than those of the control group [19].

### 3.3. Experimental Results

**Figure 7:**
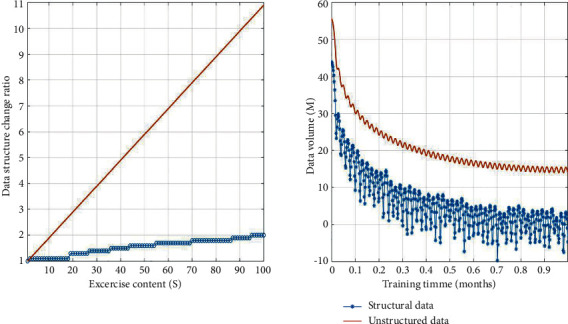
Composition of the data set.

The data set constructed in this paper is composed of strength training, aerobic training, and anaerobic training. All the data conform to the distribution, and the maximum number of iterations is set to 50. The corresponding data distribution is shown in Figure 7.

Through comparative analysis, we can see that the ratio of strength training, aerobic training, and anaerobic training to improve vocal training methods is reasonable, and the influence on vocal training is concentrated, which can meet the requirements in a short time. In addition, the content of the exercise is not affected by the complexity, and the influence on vocal training is strong. The reason is that the improved vocal training method adopts factor Cauchy(0,1) adjustment to sports training content and chooses different training strategies, which makes the training results more in line with the requirements [20].

**Figure 8:**
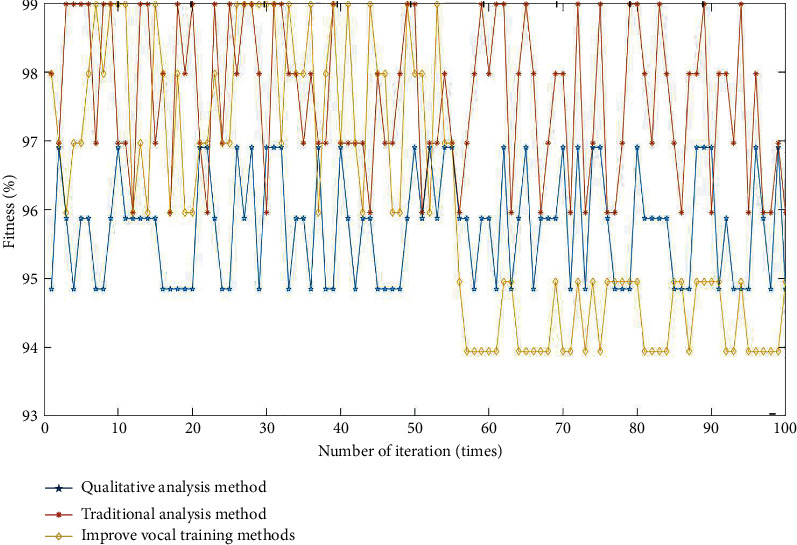
Comparison of fitness schemes of different methods.

To further prove the effectiveness of the algorithm proposed in this paper, other comparison models are introduced for comparative analysis: (1) traditional training method; (2) qualitative analysis method. The comparison results are shown in Figure 8.

As can be seen in Figure 8, the accuracy of the vocal training method in this paper is the highest. Under the same complexity, the stability of voice training results is the highest, followed by traditional analysis methods and finally qualitative analysis methods [21]. Monte Carlo combined dynamic factor algorithm data fluctuation range is small, and the analysis is relatively stable and does not appear more than 30% of the floating change. Compared with the Monte Carlo combined dynamic factor algorithm, the traditional algorithm changes more than 30%, and the frequency of change significantly increased. The reason is that the Monte Carlo algorithm reduces the complexity of sample data, provides corresponding optimization strategies for different training results, and improves the accuracy of results, which is consistent with the related research by Shyamal Kumar and Srivastava [22]. For different training indicators, the results of different analysis methods are shown in Table 5.

From Table 5, we can see that the accuracy of the improved vocal training method proposed in this paper has not changed with the change in sports content. The main reason is that the dynamic analysis of exercise content by adjusting factors and adopting different optimization strategies can adapt to the change of exercise content more flexibly. Therefore, the Monte Carlo algorithm can not only reduce the impact of motion content on the results but also quickly and accurately analyze different motion content.

## 4. Conclusion

According to the theory of vocal music, this paper proposes an improved vocal training method based on the Monte Carlo algorithm and clustering algorithm [23]. By setting the score of vocal evaluation, vocal training standards, and dynamic reference strategies, the vocal training is optimized. The vocal training method constructed in this paper firstly classifies the sample set discretely [24] and then calculates the training results. MATLAB simulation results show that the improved vocal training method constructed in this paper has higher accuracy and better convergence, meets the requirements of different sports exercises, and analyzes vocal training more objectively. Through the above analysis, it can be seen that the Monte Carlo algorithm combined with the dynamic factor algorithm can realize the optimization of vocal training, and the analysis results are stable and accurate, which is significantly better than the traditional view. Monte Carlo algorithm has high intelligence, which can realize a large number of data analyses and meet the actual requirements of training. However, there are some deficiencies in the research on the selection of exercise content. Future research will analyze this aspect and analyze the vocal training results more accurately.

## Figures and Tables

**Table 1:** Contents of vocal training for college students.

Type	Exercise		Vocal training	
	Exercise time (months)	Exercise content (*s*)	Breath (ml)	Muscle strength (*N)*
Folk music	4.2	31	16.11	4.73
Popular	3.2	71	18.32	7.96
Jazz	3.5	23	31.11	2.68
Rock and roll	5.2	12	22.38	2.58
Opera	3.2	22	19.13	1.13
Others	4.2	81	29.15	1.98

**Table 2:** Evaluation of traditional training results.

Indicators	Evaluation criteria	Score	Exercise combination
*x*_*11*_	*w1*	*λ*	*d*_*1*_
*x*_*22*_	*w2*	*λ*	*d*_*2*_
*…*	*w3*	*λ*	…
*xnn*	*wn*	*λ*	*dn*

**Table 3:** Judgment results of different functions.

Function	Sports content	Content quantity	Optimal scheme number	Number of policies	Optimization success degree	Degree of conformity with standards	Standard number
Breath	Running	3	2	3	90.2	95.4	4
	Skipping rope	2	2	7	92.3	92.6	2

Coordination	Push-ups	3	3	4	95.7	98.9	3
	Sit-ups	2	2	9	91.8	95.3	4

Strength	Shot put	2	1	5	89.3	94.7	2
	Shot put	4	2	4	85.7	96.8	3

Final result	Javelin	2	1	3	92.4	92.4	11
	Long jump	3	3	6	93.6	90.1	4

**Table 4:** Collection of the types and proportion of exercise content.

Group	Training content	Training score	Training results
The improved method proposed in this paper	Bel canto	23 ± 1.12^*∗*^	Excellent
	Folk music	12 ± 0.23^*∗*^	Excellent
	Popular	81 ± 0.47^*∗*^	Excellent

Traditional training method	Bel canto	22 ± 0.72	Good
	Folk music	9 ± 0.42	Good
	Popular	73 ± 0.21	Good

Note: compared with traditional training methods, ^*∗*^*P* < 0.05.

**Table 5:** Recognition accuracy of different analysis methods.

Scheme type	Improved vocal training methods		Traditional analysis method		Qualitative analysis method	
	Breath	Strength	Breath	Strength	Breath	Strength
Strength type	96.91	97.94	94.85	95.88	98.97	96.91
Aerobic type	91.03	95.88	92.78	93.81	97.94	91.03
Anaerobic type	93.81	80.03	96.91	90.00	91.03	93.81
Comprehensive type	97.94	91.03	95.88	90.00	95.88	97.94

## Data Availability

The data used to support the findings of this study are available from the corresponding author upon request.
